# Antibacterial and Antioxidant Compounds from the Flower Extracts of *Vernonia amygdalina*

**DOI:** 10.1155/2018/4083736

**Published:** 2018-03-20

**Authors:** Abere Habtamu, Yadessa Melaku

**Affiliations:** Chemistry Department, Applied Natural Sciences, Adama Science and Technology University, Adama, Ethiopia

## Abstract

*Vernonia amygdalina* is traditionally used in Ethiopia to treat various diseases. This prompted us to isolate bioactive compounds from the flowers of this plant. The CHCl_3_ extract after silica gel column chromatography has led to the isolation of two compounds identified as tricosane **(1)** and vernolide **(2)**, while the acetone extract furnished isorhamnetin **(3)** and luteolin **(4)**. The acetone extract and isorhamnetin significantly scavenged the 2,2-diphenyl-1-picrylhydrazyl (DPPH) radical by 91.6 and 94%, respectively. It was also shown that the acetone extract and isorhamnetin inhibited lipid peroxidation by 74 and 80%, respectively. The extracts and isolated compounds were also evaluated for their antibacterial activity with the CHCl_3_ extract and vernolide showing strong activity against *S. aureus* with an inhibition zone of 21 and 19 mm, respectively. On the other hand, the acetone extract and isorhamnetin were active against all bacterial pathogens tested. The work presented herein has demonstrated that vernolide and isorhamnetin had antibacterial activity. The antioxidant activity displayed by the flowers of *V. amygdalina* is accounted to the presence of isorhamnetin. Therefore, the biological activities displayed by the extracts and isolated compounds from this plant corroborate the traditional uses of this plant by the local people against various diseases.

## 1. Introduction

*Vernonia amygdalina* Del. is a soft woody shrub or tree belonging to the family Asteraceae and genus *Vernonia* [1]. The plant is predominantly found in Africa. It is a perennial plant characterized by its bitter sap from the leaf which has been widely explored for its medicinal use. This plant grows to 10 m tall with petiole leaf of about 6 mm in diameter and elliptic in shape [2]. In the Ethiopian highland, *V. amygdalina* has been classified by the farmer as a multipurpose fodder tree with high biomass yield, easy propagation, high adaptability, and high compatibility with other crops which do not compete with them for soil nutrients or moisture but instead help to improve the soil fertility and growth of perennial crops [3]. *V. amygdalina*, locally called *ebicha* in Afan Oromo and *Grawa* in Amharic (Ethiopia), is quite commonly used in Ethiopia in the preparation of local beer and also as fumigant. It is also used as fire wood [4].

*V. amygdalina* is traditionally used to treat many ailments including diabetes [5, 6], antihelminth, antimalarial, laxative, digestive tonic, appetizer, and febrifuge [7]. In some African countries including Ethiopia, *V. amygdalina* is among medicinally significant plants used against malaria, helminth infections, gastrointestinal disorders, and fever [8]. The species is also used to promote wound healing [9] and to treat microbial infections [10]. The main bioactive constituents of the leaves were reported as sesquiterpene lactones [11]. Some of them include vernonioside A1, vernonioside A2, vernonioside B1, vernonioside B2 [12, 13], vernodalin, vernolepin, vernomygdin, vernodalol, and vernodalinol [14, 15]. Despite numerous report on the secondary metabolite profile of the leaves, to the best of our knowledge there is no prior scientific report on the chemical constituents of the flowers of this species. Furthermore, the antioxidant and antibacterial activities of the flower extracts and constituents of *V. amygdalina* have not been studied. Hence, in this paper, we report the results of our investigation of effects of the extracts and constituents of the flowers of *V. amygdalina* on *E. coli*, *K. pneumonia*, *P. mirabilis*, *S. aureus*, and *S. bacillus*. The results of the evaluation of the radical scavenging activities and antilipid peroxidation potentials of the flower extracts and constituents of *V. amygdalina* are also described herein.

## 2. Materials and Methods

### 2.1. Plant Material

**Figure 1:**
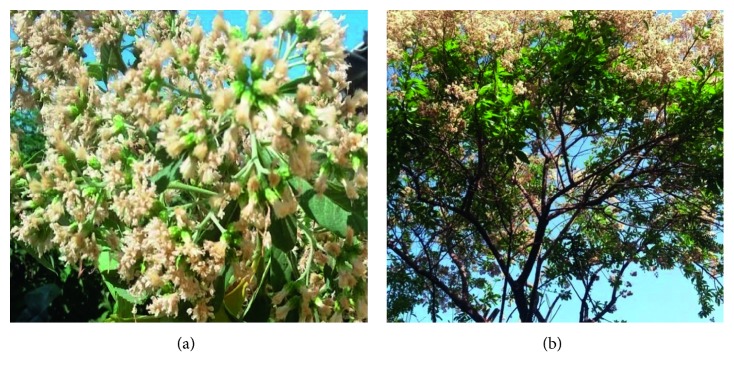
Flowers (a) and aerial part (b) of *Vernonia amygdalina* from Wonji, Ethiopia, on February 12, 2017 (picture taken by Abere Habtamu).

The flowers of *V. amygdalina* were collected on February 12, 2017 from Wonji district, which is located at 107 km Southeast of Addis Ababa, Ethiopia. Identification and authentication of the plant specimen was done at the National Herbarium of Addis Ababa University by Mr. Melaku Wondafrash and voucher specimen (AB002) was deposited (Figure 1).

### 2.2. Extraction and Isolation

The air-dried powdered flowers of *V. amygdalina* (500 g) were successively extracted with hexane (2 L), chloroform (2 L), and acetone (2 L) each for 24 hours at room temperature with frequent agitation on the automatic agitator. It was filtered and concentrated to furnish 2.2 g (0.4%), 8.9 g (1.78%), and 9.4 g (1.91%), respectively.

The chloroform extract (4.5 g) was adsorbed in an equal amount of silica gel and fractionated over silica gel (160 g) column chromatography. The column was eluted with hexane : ethyl acetate : methanol of increasing polarities to furnish 14 fractions. A volume of 100 mL each was collected. The first fraction was collected with 100% hexane. F2 to F10 were eluted with hexane : EtOAc with the ratio 9 : 1, 4 : 1, 7 : 3, 7 : 3, 3 : 2, 3 : 7, 1 : 4, 1 : 4, and 1 : 9, respectively. The next two, F11 and F12, were collected using 100% EtOAc. The column was ended after collecting F13 and F14 using EtOAc : MeOH with ratio 9 : 1 and 6 : 4, respectively. F1 (22 mg) and F10 (77 mg) were shown to have a single spot on TLC and hence were analyzed with NMR, UV, and IR.

Likewise, the acetone extract (5 g) was adsorbed and fractionated over silica gel (180 g) column chromatography to furnish 15 fractions. In each case, a volume of 100 mL was collected. The first four fractions were collected using CHCl_3_. F5–F10 were collected using CHCl_3_ : MeOH with the ratio 95 : 5, 9 : 1, 9 : 1, 9 : 1, 85 : 15, and 4 : 1, respectively. The last five fractions were collected using CHCl_3_ : MeOH (2 : 3). F9 (70 mg) was shown to have one spot on TLC and hence was analyzed with NMR, UV and IR.

### 2.3. Antibacterial Activities

The antibacterial activities of the extracts and constituents of the flower of *V. amygdalina* were checked using agar well diffusion method [16] against five bacterial strains, three Gram negative (*Escherichia coli*, *Klebsiella pneumoniae*, and *Proteus mirabilis*) and two Gram positive (*Staphylococcus aureus* and *bacillus*). Bacterial cultures were maintained on nutrient Muller–Hinton agar at 37°C, and the cultures were kept in appropriate media slants and stored at 4°C until used. Colonies of bacteria (24-hour old culture) were diluted by physiological normal saline (0.85%), to make a 0.1 McFarland standard suspension, and then the bacteria was inoculated into sterile Petri dishes of 60 mL of Muller–Hinton agar plates. The plates were shaken gently to allow evenly mixing of bacterial cells and agar. All samples were dissolved in methanol to furnish 10 mg/mL. From each sample, 100 *µ*L of each concentration saturated with discs (6.00 mm diameter disc) was placed on plate and incubated at 37°C for 24 hours. The diameters of the inhibition zones were calculated. Clear inhibition zones formed around the discs indicated the presence of antibacterial activity [16]. Control wells containing neat solvents (negative control) and chloramphenicol (positive control) were also run parallel in the same plate.

### 2.4. Antioxidant Assay

The antioxidant activities of the extracts and constituents were studied using DPPH and ferric thiocyanate methods.

#### 2.4.1. DPPH Radical Scavenging Assay

The radical scavenging assay of the extract and constituents of the flowers of *V. amygdalina* was assessed using DPPH according to the following procedure [17]: the CHCl_3_ extract was dissolved in methanol to afford 1 mg/mL. It was serially diluted in methanol to give concentration of 500, 250, 125, and 62.5 *µ*g/mL. To 1 mL of each concentration, 4 mL DPPH (0.04% DPPH in MeOH) was added to make 100, 50, 25, and 12.5 *µ*g/mL solutions. This was repeated for the acetone extract and pure constituents. Then, all the samples prepared were incubated in an oven at 37°C for 30 min and then absorbance was recorded at 517 nm using a UV-Vis spectrophotometer. The experiments were performed in triplicates. The percentage inhibition was calculated using the following formula:

(1) $$\begin{array}{c} \%\;\;\mathrm{inhibition}\;=\;\frac{\left(A_{\text{control}}\;\;-\;\;A_{\text{extract}}\right)}{A_{\text{control}}}\;\times \;100, \end{array}$$

where *A*_control_ is the absorbance of DPPH solution and *A*_extract_ is the absorbance of the test sample (DPPH solution plus sample).

#### 2.4.2. Ferric Thiocyanate Method

The antioxidant potential of the extracts and constituents of the flowers of *V. amygdalina* was also assessed according to the method of Nagatsu [18]. Each 0.1 mg of the extracts and pure compounds of *V. amygdalina*, 100 *µ*L of linoleic acid, EtOH (5 mL), and phosphate buffer (5 mL, 0.05 M, pH = 7) in water were separately added into a vial and incubated at 40°C in an oven. After 24 h, 0.1 mL from each were taken and added into a vial containing 75% aqueous EtOH (7 mL), 30% of NH_4_SCN (0.15 mL), and 0.15 mL of 0.02 M FeCl_2_ in 3.5% HCl. Each was then subjected to UV-Vis spectrophotometery to record the absorbance at 500 nm. Absorbance of the blank and ascorbic acid were done in the same fashion. The experiments were performed in triplicates. The percentage inhibition using ferric thiocyanate method is calculated according to the following formula:

(2) $$\begin{array}{c} \text{Percentage}\;\;\mathrm{inhibition}=100\;-\;\left(\frac{\text{As}}{\text{Ab}}\times 100\right)\%, \end{array}$$

where As is the absorbance of the sample and Ab is the absorbance of the blank [19].

## 3. Results and Discussion

**Figure 2:**
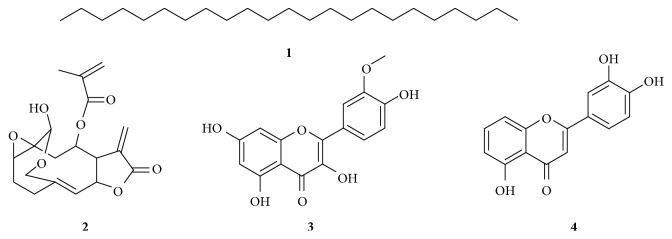
Compounds isolated from the flower extracts of *V. amygdalina*.

Four compounds were isolated and characterized from the flower extracts of *V. amygdalina* (Figure 2).

Compound **1** (22 mg) was obtained as a white power from the CHCl_3_ extract of the flower of *V. amygdalina*. The ^1^H-NMR spectrum of **1** exhibited signal at *δ* 0.9 (6H, *t*) accounted to the presence of terminal methyl group. A broad singlet observed at *δ* 1.28 (42H, *br. s*) is a characteristic of many overlapping protons on methylene carbons. The proton decoupled ^13^C-NMR spectrum of **1** with the aid of DEPT-135 suggested the presence of twenty-three carbons of which the signal observed at *δ* 14.1 is evident for the presence of terminal methyl group. The remaining carbons are all methylenes. The NMR spectral data generated for compound **1** agreed well with tricosane (Figure 2). This compound has not yet been reported from the genus.

Compound **2** (77 mg) was obtained as yellow solid from the chloroform extract of the flower of *V. amygdalina*. It was eluted with hexane : ethyl acetate (1 : 9). The ^1^H-NMR spectral data of compound **2** (Supplementary Material 1) showed the presence of exomethylene *δ*-lactone at *δ* 6.3 (1H, *d*) and *δ* 5.9 (1H, *d*). Other diastereotopic methylene protons were evident at *δ* 6.1 (1H, *d*) and 5.7 (1H, *d*). Another olefinic methine proton is observed at *δ* 5.5 (1H, *d*). The signal at *δ* 5.2 (1H, *t*) is due to methine proton on oxygenated carbon. Methylene protons on oxygenated carbons are evident at *δ* 4.6 and 3.7 (2H, *d*, H-15). The signal at *δ* 4.5 (1H) indicated that the presence of methine proton on carbon bearing two oxygens. Moreover, the spectral data of this compound showed one methyl group on quaternary carbon at *δ* 1.9 (3H, *s*). The ^13^C-NMR spectral data (Supplementary Material 2) displayed the presence of nineteen carbon resonances which agreed well with literature reported from vernolide [20] whose structure is shown in Figure 2. The structure was further confirmed with extensive 2D-NMR including COSY, HSQC, and HMBC (Supplementary Materials 4–6).

Compound **3** (70 mg) was isolated from the acetone extract of the flower of *V. amygdalina*. It was eluted using CHCl_3_ : MeOH (85 : 15) as an eluent and visualized as a yellow spot after spraying with vanillin in H_2_SO_4_. Analysis of the proton NMR spectrum of compound **3** (Supplementary Material 7) showed signal at *δ* 12.7 (1H, *s*) due to the chelated hydroxy group which is evident for the presence of 5-OH. The three aromatic proton signals at *δ* 7.5 (1H, *d*, *J* = 2.00 Hz, H-2′), 7.6 (1H, *dd*, *J* = 8.4 Hz and 2.00 Hz, H-6′), and 6.9 (1H, *d*, *J* = 8.4 Hz, H-5′) are typical of flavonoid with 3′,4′-disubstituted B-ring. The other aromatic proton signals at *δ* 6.4 (1H, *d*, *J* = 2 Hz, H-8) and 6.2 (1H, *d*, *J* = 2 Hz, H-6) are apparently due to *meta* coupled protons on the A-ring of flavonoid. The presence of one methoxy group is evident at *δ* 3.8 (3H, s). The presence of *α*, *β*-unsaturated ketone is evident from the appearance of the carbonyl carbon signal at *δ* 178.3. Other signals in the ^13^C-NMR spectrum (Supplementary Material 8) were observed at *δ* 164.5 (C-7), 161.7 (C-9), 156.7 (C-5), 156.0 (C-2), 149.1 (C-4′), 145.6 (C-3′), 138.0 (C-3), 121.2 (C-1′), 121.0 (C-6′), 116.1 (C-5′), 115.8 (C-2′), 104.6 (C-10), 98.9 (C-6), and 94.0 (C-8). The carbon resonance at *δ* 60.0 is due to the presence of the methoxy group. Based on the above spectral data, compound **3** was identified as isorhamnetin whose structure is shown in Figure 2.

Compound **4** was isolated from the acetone extract of the flower of *V. amygdalina*. It was eluted using CHCl_3_ : MeOH (4 : 1) as an eluent and visualized as a yellow spot after spraying with vanillin in H_2_SO_4_. The ^13^C-NMR spectrum of **4** with the aid of DEPT-135 revealed the presence of fifteen carbon resonances which agreed well with the literature reported for luteolin (Figure 2).

### 3.1. Antibacterial Activity

The antibacterial activities of the hexane, chloroform, acetone extract, and pure constituents of the flowers of *V. amygdalina* were investigated using agar well diffusion method, against the selected human pathogens such as *E. coli*, *K. pneumoniae*, *P. mirabilis*, *S. aureus*, and *S. bacillus*. The results are presented in Table 1.

**Figure 3:**
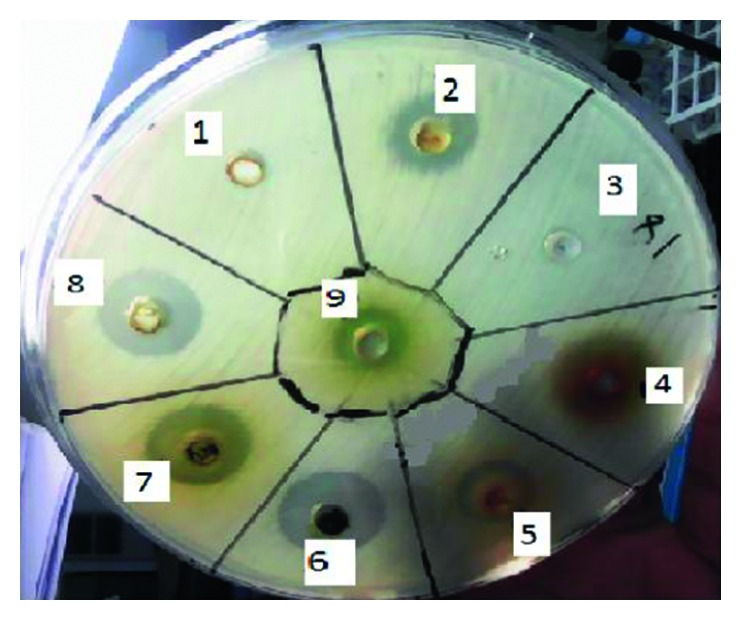
Zone of inhibition of constituents of the flowers of *V. amygdalina* against *S. aureus*. Note: 1 and 3 are inhibition zones exhibited by DMSO while 4 represents chloramphenicol; 2, 6, 7, 8, and 9 represent inhibition zone displayed by the hexane extract, chloroform extract, acetone extract, isorhamnetin, and vernolide at 1 mg/mL; 5 is hexane extract at 0.5 mg/mL.

The extracts and isolated compounds showed considerable difference in antibacterial activities against all selected bacteria's with zone of inhibition ranging from 6 to 21 mm (Table 1). The acetone and chloroform extracts of the flowers of *V. amygdalina* demonstrated pronounceable activity against *S. aureus* (Figure 3) compared to chloramphenicol used as a standard antibiotic. This is in conformity with the result reported for the leaf extract of *V. amygdalina* against *S. aureus* [21]. The activity displayed by the chloroform extract is likely accounted to the presence of vernolide which showed an inhibition zone of about 19 mm. This is significantly higher than the activity displayed by chloramphenicol. Vernolide exhibited relatively better activity against Gram-positive bacteria with maximum inhibition zone (19 mm) observed for *S. aureus*. This is in close agreement with the literature reported for same compound [20]. The zone of inhibition displayed by the constituents of the flowers of *V. amygdalina* is depicted in Figure 3. The chloroform extract was found insensitive against *E. coli*, *K. pneumonia*, *P. mirabilis*, and *S. bacillus*. On the other hand, the acetone extract displayed broad range of antibacterial activities against all five strains tested in this study. Similar effects have been reported for the leaves extract of *V. amygdalina* against *E. coli* [22]. The wide zone of inhibition of the acetone extract of *V. amygdalina* showed that it had great potential as a remedy for infections/diseases caused by bacterial pathogens. Isorhamnetin isolated in this work from this plant showed modest activity with inhibition zone ranging from 9 to 14 mm against all bacterial strains. The work presented herein has demonstrated that the acetone extract, vernolide, and isorhamnetin identified in the flowers of *V. amygdalina* had antibacterial activity.

### 3.2. Antioxidant Activity

#### 3.2.1. DPPH Radical Scavenging Assay

DPPH radical scavenging assay is a simple method for finding antioxidants by measuring absorbance at 517 nm due to the stable 2,2-diphenyl-1-picrylhydrazyl (DPPH) radical [19]. Though their degree varies, the extracts and pure constituents of the flowers of *V. amygdalina* are able to reduce the stable DPPH radical to the yellow-colored diphenylpicrylhydrazicine indicating their potential as radical scavengers. The acetone extract reduced DPPH radicals significantly as compared to the chloroform extract, with the earlier inhibiting the radical by 91.46% at 100 *µ*g/mL (Table 2). This is evident from the low IC50 (42 *µ*g/mL) value observed for the acetone extract. At the same concentration standard, ascorbic acid scavenged the DPPH radical by 97.7%. Plant phenolics are a major group of compounds acting as primary antioxidants or free radical scavengers [23]. Therefore, the observed high free radical scavenging activity of the acetone extract is accounted to the presence of two natural antioxidants, isorhamnetin and luteolin, identified in this study from the flowers of this species. The radical scavenging potential of isorhamnetin was found to be 94%, which is comparable to ascorbic acid used as the positive control. Both isorhamnetin and luteolin are flavonoids with free hydroxyl group which can donate hydrogen and electron and hence responsible for the radical scavenging activities of the flower of *V. amygdalina*. This agrees well with the literature reported for the antioxidant activity of luteolin [23].

#### 3.2.2. Ferric Thiocyanate Method

The degree of lipid peroxidation which was evaluated using ferric thiocyanate method can be used to measure the antioxidant potential of compounds or extracts.  Table 3 shows the results of the antilipid peroxide formation of the flower extracts and constituents of *V. amygdalina*.

As depicted in Table 3, the acetone extract and isorhamnetin inhibit peroxide formation by 74 and 80%, respectively, demonstrating their potential in preventing the formation lipid peroxides. The results were turned out to be comparable with ascorbic acid with inhibition of 83%. On the other hand, the chloroform extract and vernolide were shown to have low ability of inhibiting peroxide formation compared with the natural antioxidant. This indicates that the antioxidant compound of the flowers of *V. amygdalina* resides in the acetone extract with the main active ingredient found to be isorhamnetin.

## 4. Conclusion

In conclusion, the work presented herein has demonstrated that the acetone extract and vernolide had strong antibacterial activity compared to chloramphenicol. The antioxidant activities displayed by the acetone extract and isorhamnetin were significant compared with ascorbic acid indicating the potential of the flowers of this species as natural antioxidants. Therefore, biological activities displayed by the flower extracts and constituents of the flowers of *V. amygdalina* corroborate the traditional uses of this plant against various ailments including bacteria.

## Figures and Tables

**Table 1:** Inhibition zone of the extracts and pure compounds of flowers of *V. amygdalina*.

Extract/compounds/control	Zone of inhibition (mm)				
	*E. coli*	*K. pneumoniae*	*P. mirabilis*	*S. aureus*	*S. bacillus*
Chloroform extract	6	6	6	21	6
Acetone extract	10	12	11	17	12
Vernolide	10	12	6	19	12
Isorhamnetin	10	14	12	11	9
Chloramphenicol	18	18	18	18	18
DMSO (control)	0	0	0	0	0

Chloramphenicol and DMSO were used as the positive and negative controls, respectively.

**Table 2:** Radical scavenging activities of the extracts and constituents of the flowers of *V. amygdalina*.

Chloroform extract		Acetone extract	Isorhamnetin	Vernolide
Concentration (*µ*g/mL)	% DPPH inhibition	% DPPH inhibition	% DPPH inhibition	% DPPH inhibition
12.5	32.8 ± 0.2	69.0 ± 1.1	76.5 ± 0.5	24 ± 0.4
25	37.1 ± 0.9	74.5 ± 0.4	84 ± 0.6	31 ± 2.0
50	43.0 ± 0.4	83.0 ± 0.2	88 ± 0.8	37 ± 0.8
100	54.1 ± 0.5	91.6 ± 0.8	94 ± 0.1	49 ± 0.2

The results are reported as mean ± SD of three replicates. Ascorbic acid was used as the positive control with % DPPH inhibition of 97.7.

**Table 3:** Antilipid peroxidation activities of extracts and constituents of the flowers of *V. amygdalina*.

Sample name	Absorbance at 500 nm	% inhibition	Remark
Blank	0.56	—	—
Ascorbic acid	0.10	83 ± 0.1	—
CHCl_3_ extract	0.30	46 ± 0.8	—
Acetone extract	0.15	74 ± 0.3	—
Vernolide	0.35	38 ± 0.7	—

The results are reported as mean ± SD of three replicates. Ascorbic acid was used as the positive control.

## Supplementary Materials

Supplementary Materials The ^1^H-, ^13^C, DEPT-135, COSY, HSQC, and HMBC spectra generated for identifying compound **2** as vernolide are annexed as a supporting material 1, 2, 3, 4, 5, and 6, respectively. Likewise, the NMR spectra generated for characterization of isorhamnetin are annexed as supporting material 7–9. 1. 1H-NMR spectrum of vernolide. 2. 13C-NMR spectrum of vernolide. 3. DEPT-135 NMR spectrum of vernolide. 4. COSY NMR spectrum of vernolide. 5. HSQC NMR spectrum of vernolide. 6. HMBC NMR spectrum of vernolide. 7. 1H-NMR spectrum of isorhamnetin. 8. 13C-NMR spectrum of isorhamnetin. 9. DEPT 135 NMR spectrum of isorhamnetin.
