# Supplementary material for: Antibacterial and Antioxidant Compounds from the Flower Extracts of Vernonia amygdalina
Source: Adv Pharmacol Sci. 2018 Mar 20;2018:4083736. doi: 10.1155/2018/4083736 (PMC5884303; doi:10.1155/2018/4083736)

Supporting Information

1. ^1^H-NMR spectrum of vernolide


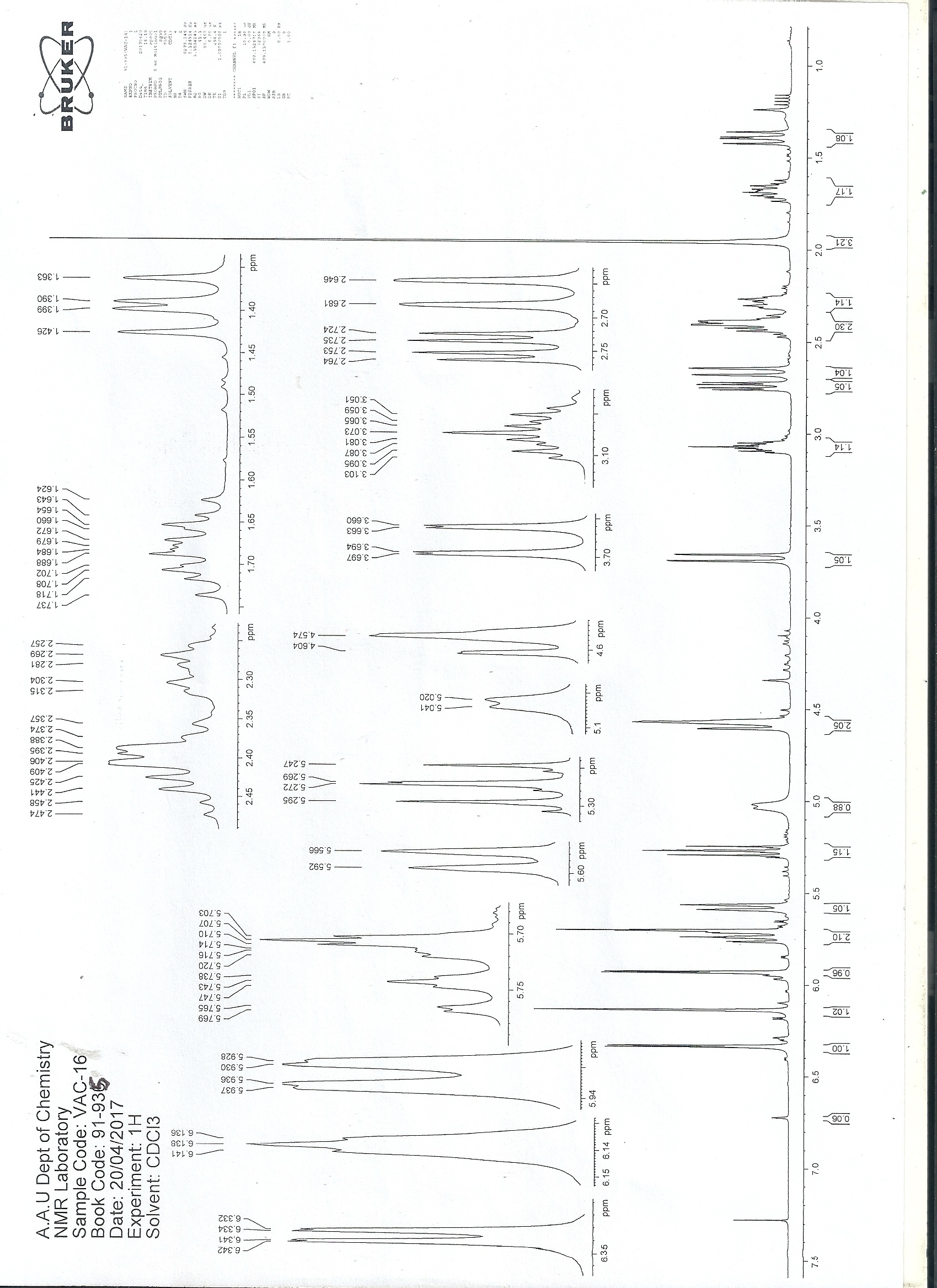


1. ^13^C-NMR spectrum of vernolide


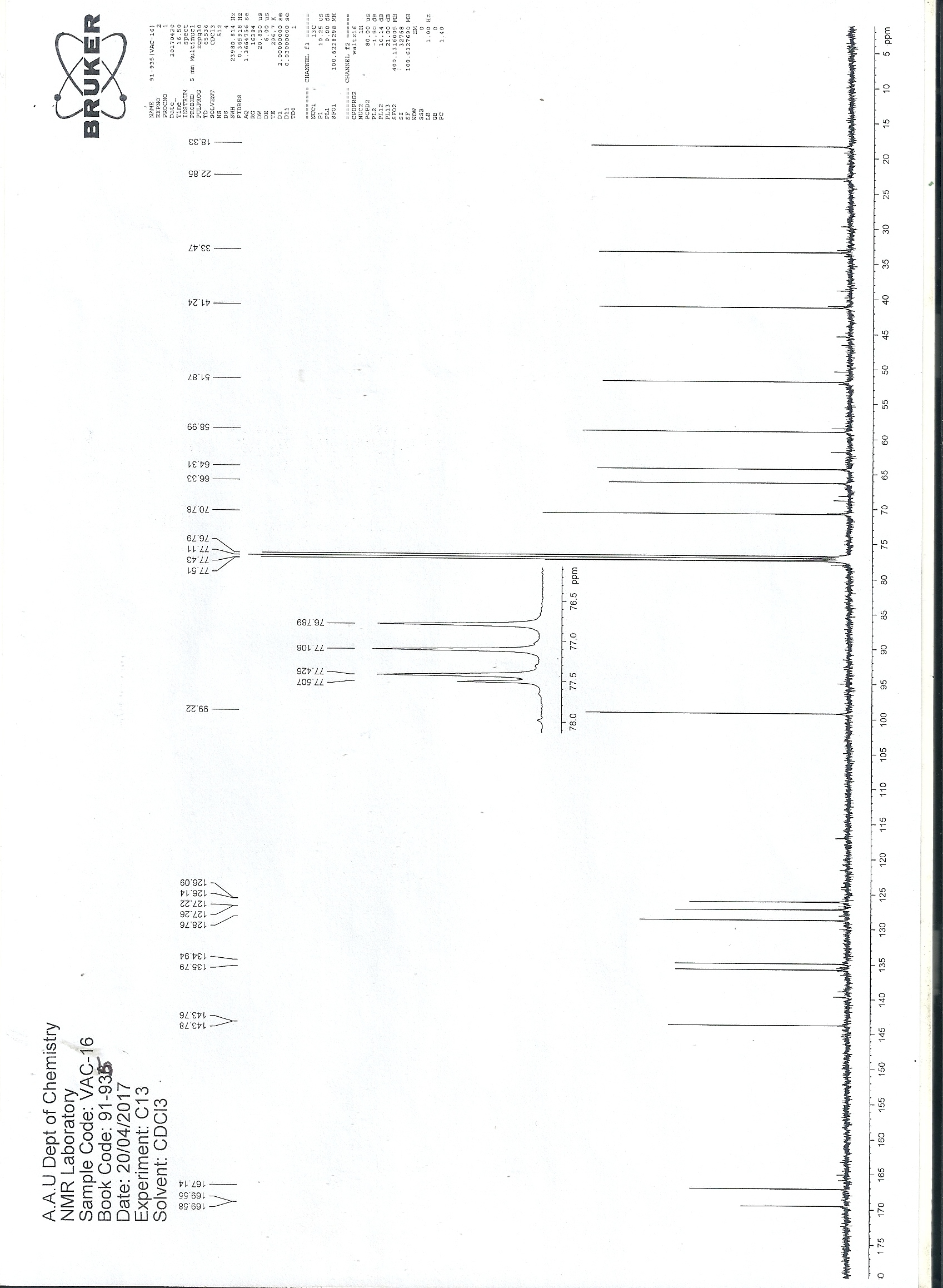


1. DEPT-135 NMR spectrum of vernolide


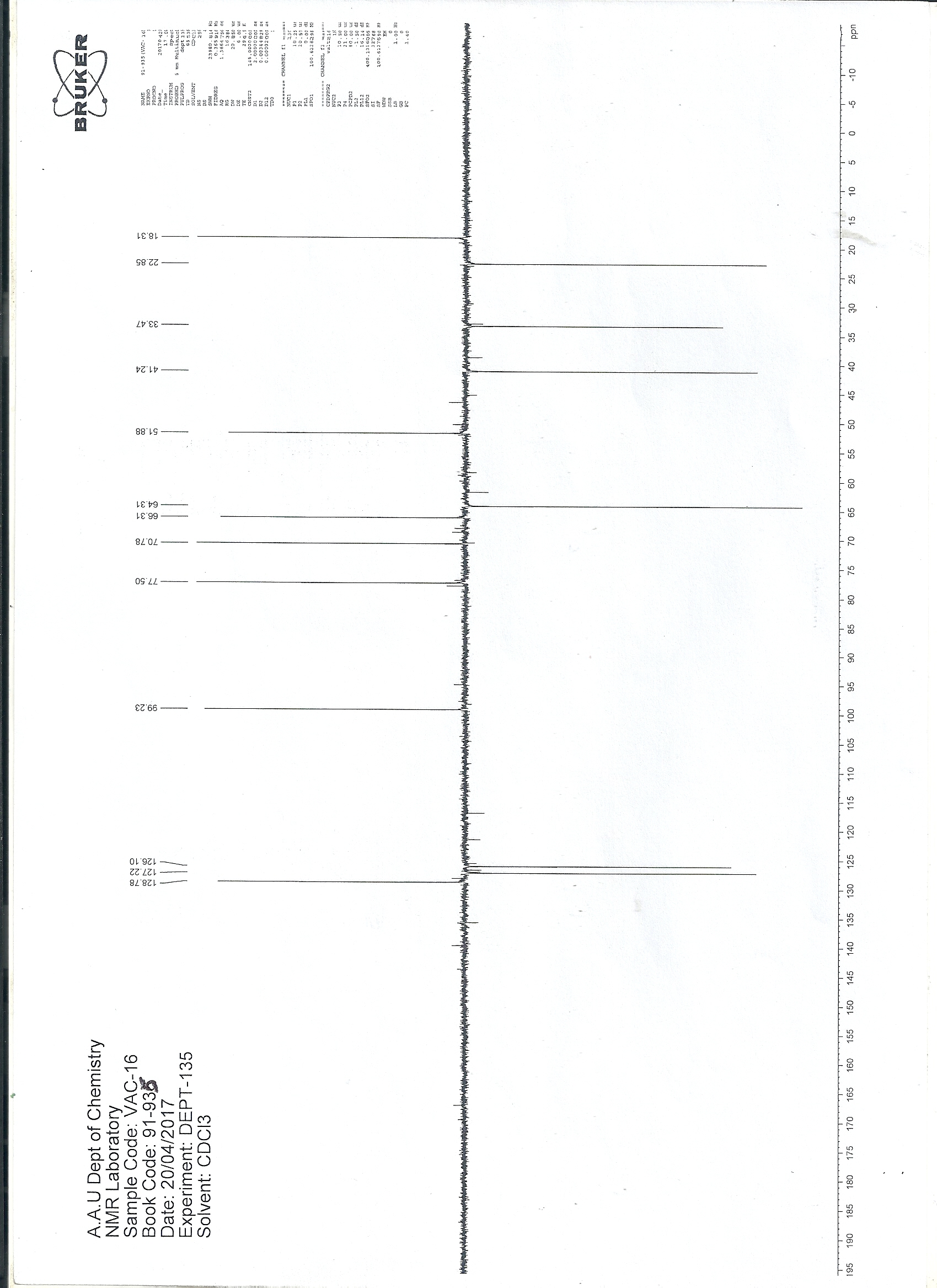


1. COSY NMR spectrum of vernolide


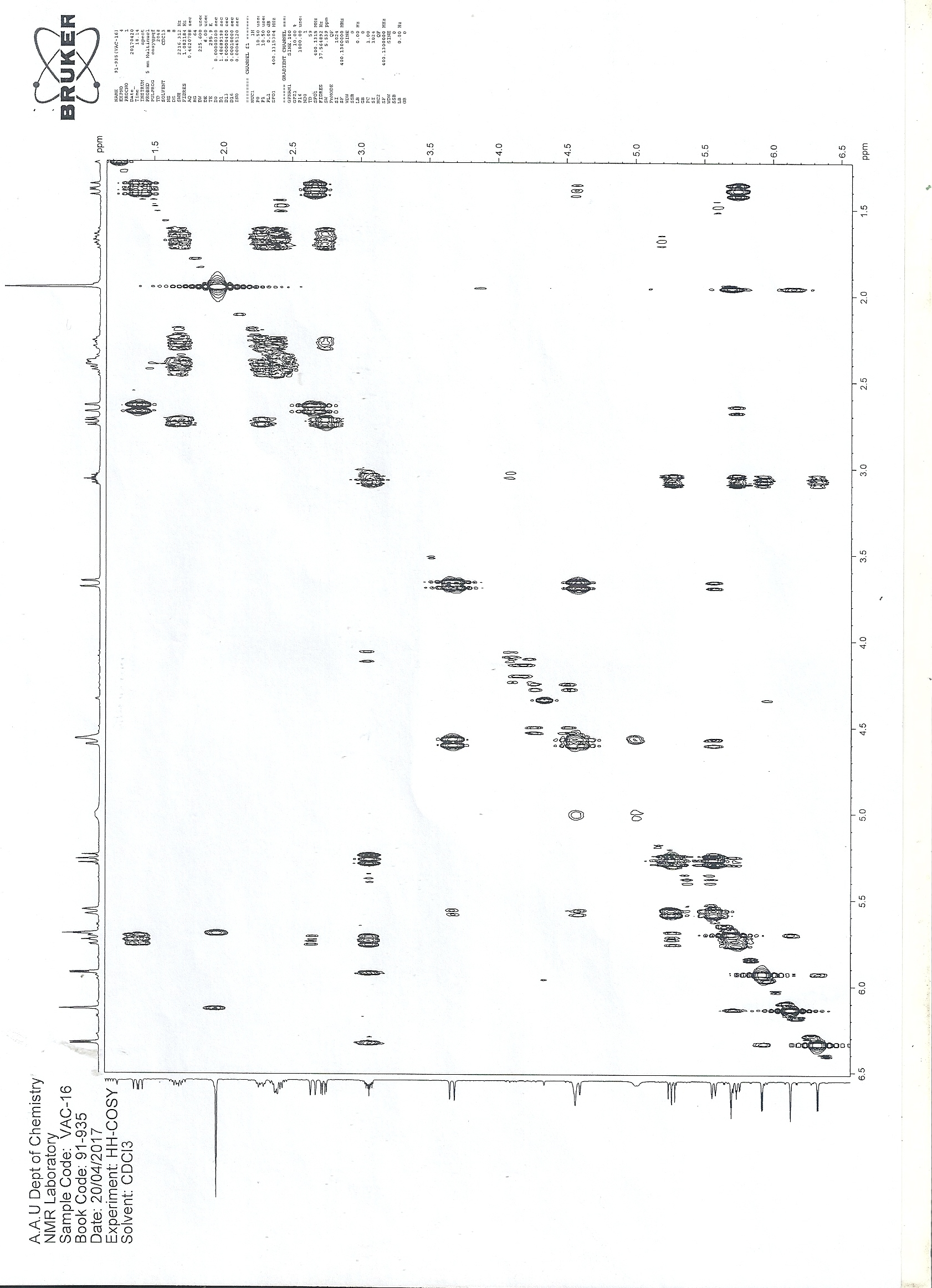


1. HSQC NMR spectrum of vernolide


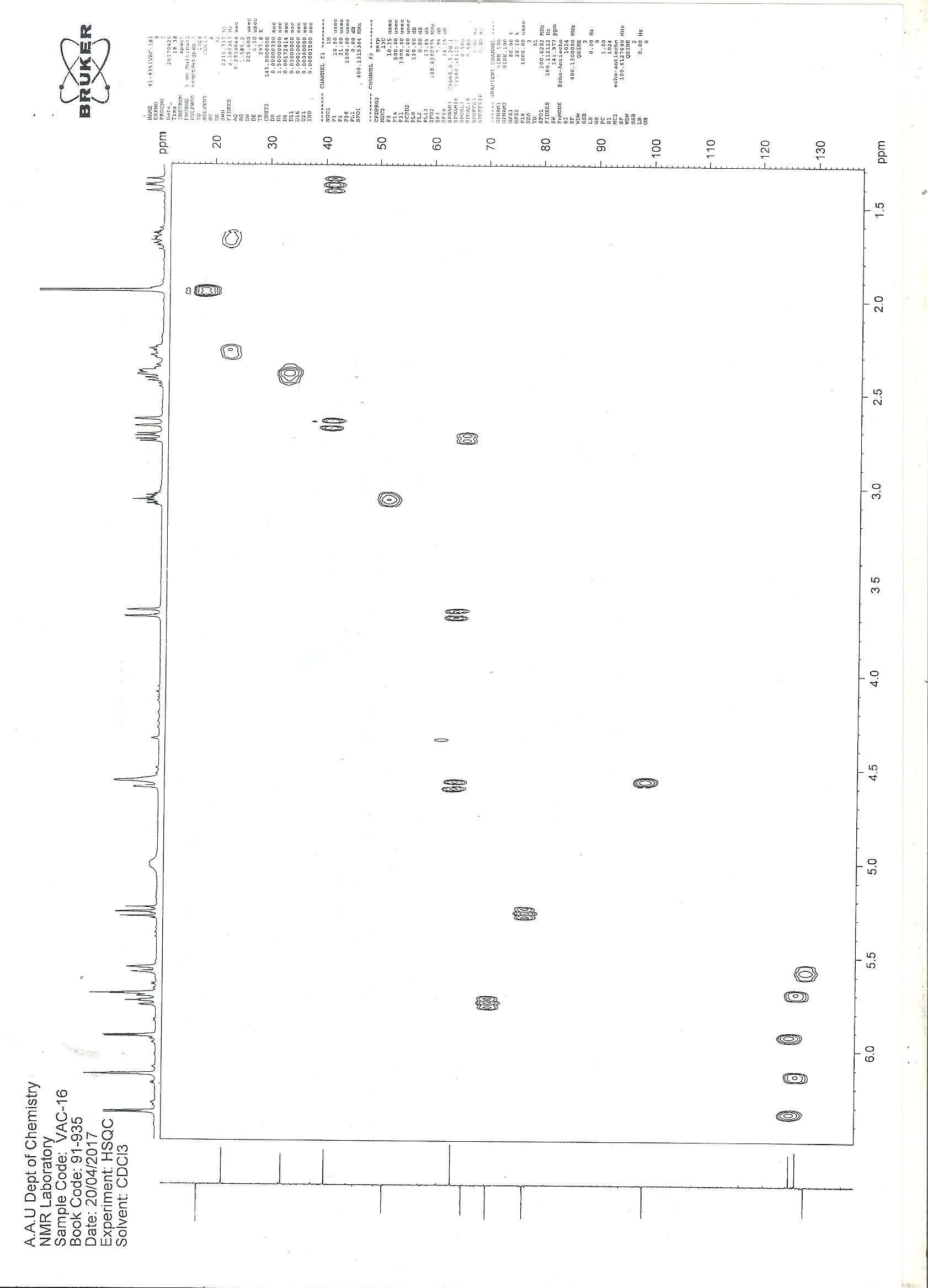


1. HMBC NMR spectrum of vernolide


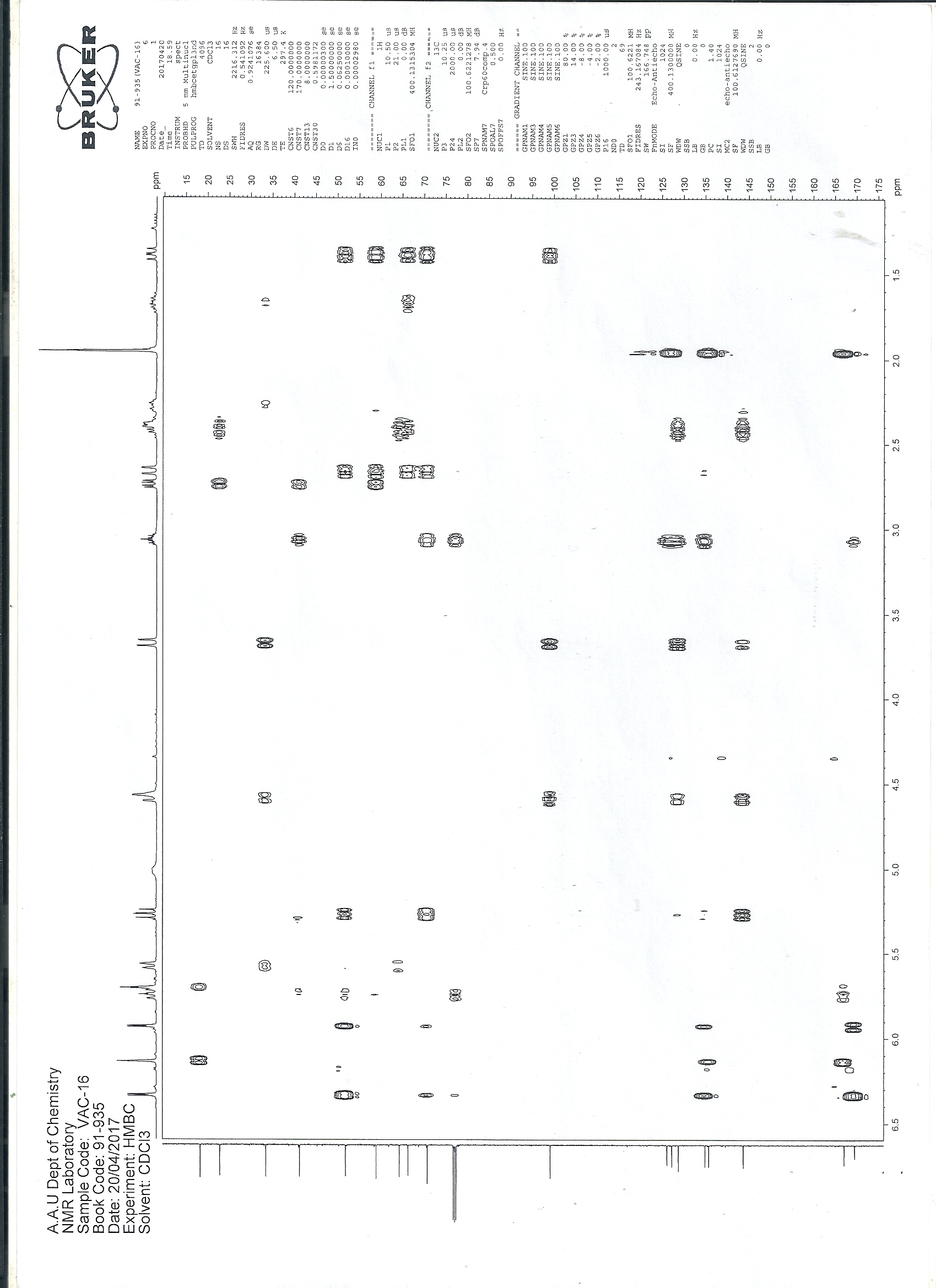


1. ^1^H-NMR spectrum of isorhamnetin


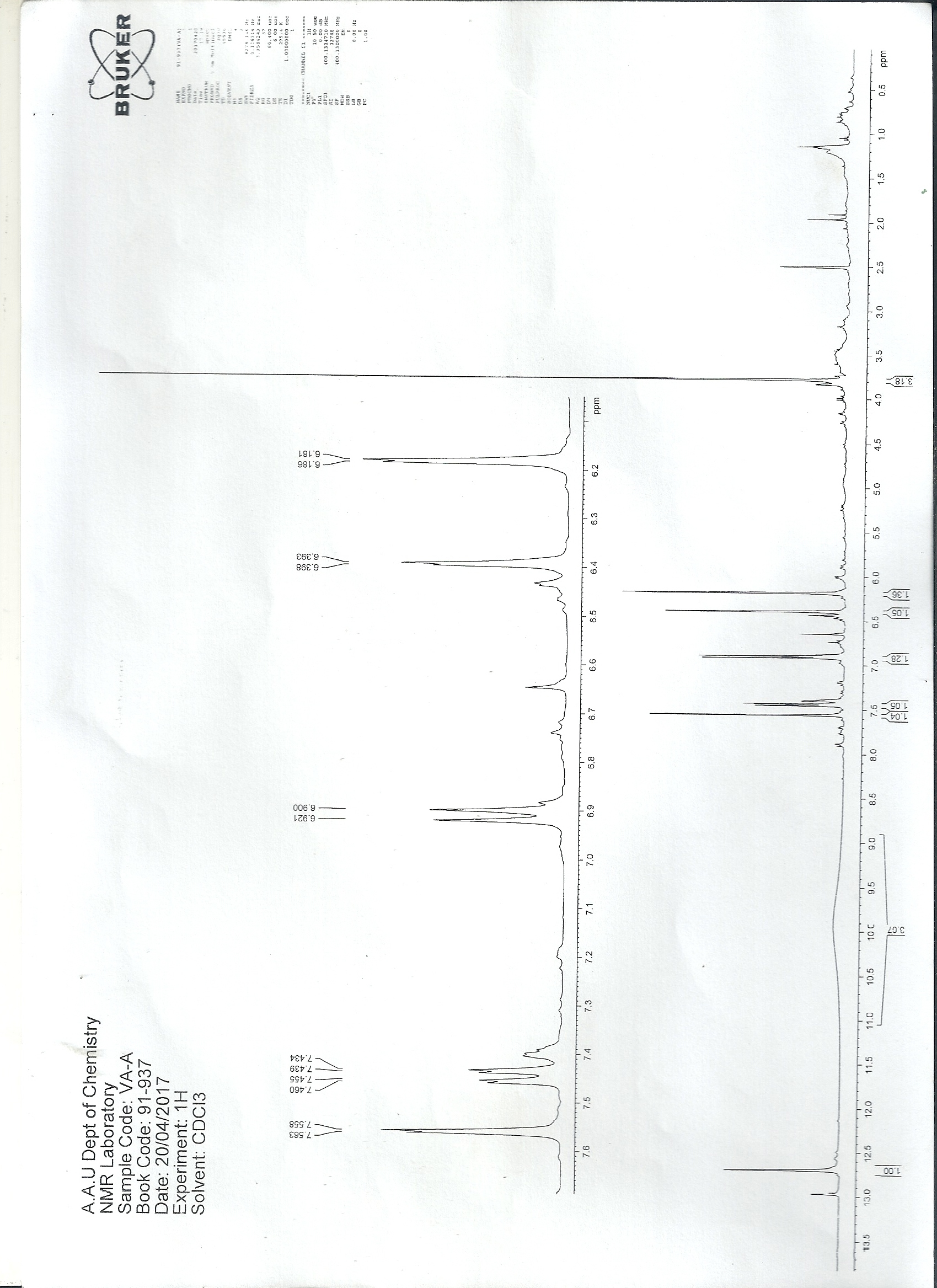


1. ^13^C-NMR spectrum of isorhamnetin


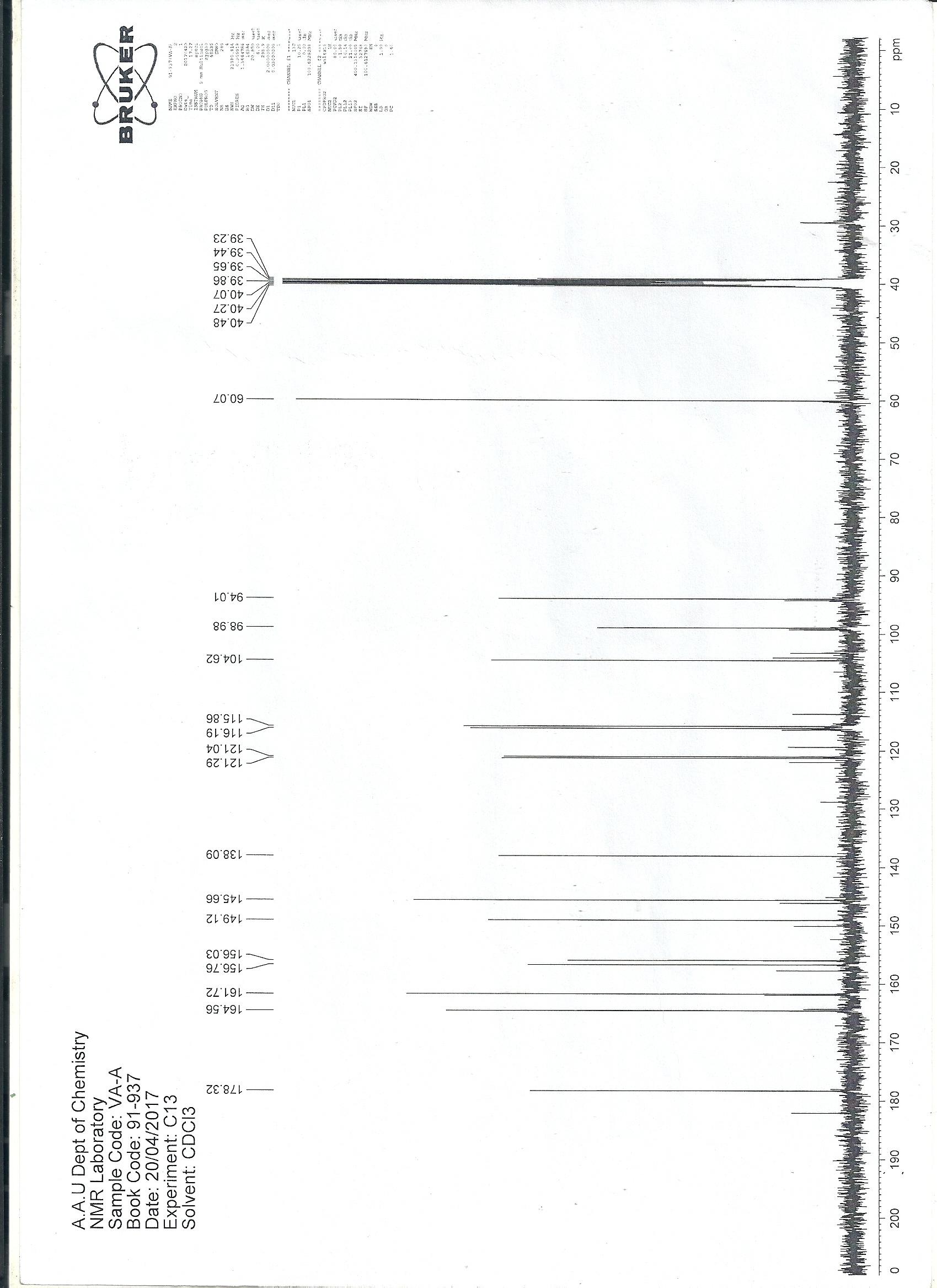


1. DEPT 135 NMR spectrum of isorhamnetin


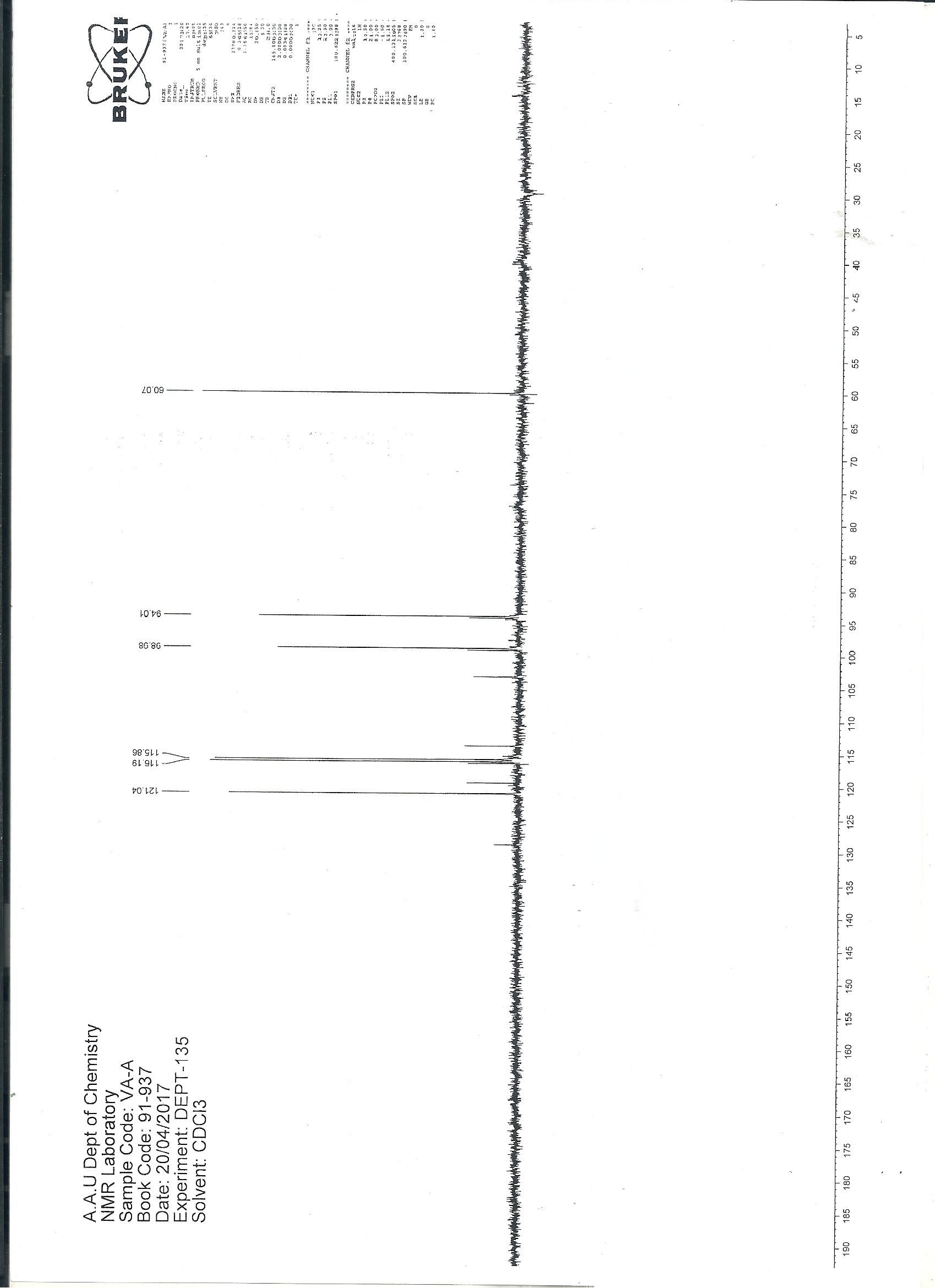

Supplement: Supplementary Materials — The 1H-, 13C, DEPT-135, COSY, HSQC, and HMBC spectra generated for identifying compound 2 as vernolide are annexed as a supporting material 1, 2, 3, 4, 5, and 6, respectively. Likewise, the NMR spectra generated for characterization of isorhamnetin are annexed as supporting material 7–9. 1. 1H-NMR spectrum of vernolide. 2. 13C-NMR spectrum of vernolide. 3. DEPT-135 NMR spectrum of vernolide. 4. COSY NMR spectrum of vernolide. 5. HSQC NMR spectrum of vernolide. 6. HMBC NMR spectrum of vernolide. 7. 1H-NMR spectrum of isorhamnetin. 8. 13C-NMR spectrum of isorhamnetin. 9. DEPT 135 NMR spectrum of isorhamnetin. [file 4083736.f1.doc]
